# 

**Published:** 1878-05

**Authors:** 


### TABLE OF CONTENTS.

### ORIGINAL COMMUNICATIONS.

PUERPERAL ECLAMPSIA. *By A Couvert, Oxford, Ga.* .................. 65

CONSERVATIVE SURGERY. *By John E. Walker, M. D., Greenesboro, Ga.* 67

VALEDICTORY ADDRESS. *By R. N. Ely, Atlanta, Ga* ................ 69

VALEDICTORY ADDRESS FOR THE GRADUATING CLASS. *By Geo. W Blanton, M. D., Dalton, Ga* .................................... 73

### REPORTS OF SOCIETIES.

Atlanta Academy of Medicine.............................. .... 79
Baltimore Medical Association .................... ............. 83
Baltimore Clinical Society.......................... .... 85

### SELECTIONS.

The Pathology of Seborrhœa, ................................ .... 89
Further Observations on Diphtheria..... ....................... 96
On the Causes of Insanity in the United States ............ ....... 98
Salicylic Acid in Lumbago........................ ........ ..... 101
On the Use of Electricity in the Treatment of Epilepsy............ 103
Duality of the Brain ........ .................................. 110
Intra-Uterine Pregnancy, complicated with Extra-Uterine Fetation—Recovery.............................................. 113
Double Fetation............... ............................ 116
Interstitial Injection of Carbolic Acid in Piles ...................... 117
A Substitute for Cod-Liver Oil in Skin Diseases.................... 118

### EDITORIAL.

Medical Association of Georgia ..................................... 120

### BIBLIOGRAPHICAL.

Atlas of Skin Diseases............................................ 123

### EXCERPTA.

Strychnine in Cough Mixtures ...................................... 124
On the treatment of Erysipelas by Silicate of Soda............, ..... 125
Arnica as a Remedy for Boils ...................................... 125
Quinine Pills.................................................... 126
Treatment of Delirium Tremens........................ ............ 127
Santonine Poisoning....................... ...................... 127
Jarborandi Proposed as a Remedy in Hydrophobia.................. 127
Chloral Hydrate as an Anticeptic.................................. 128
Constipation ..................................................... 128

